# Unveiling the Mechanisms of Bacterial Resistance and Countermeasures

**DOI:** 10.3390/pathogens14111085

**Published:** 2025-10-24

**Authors:** Yuqing Xie, Hao Lu, Yichen Liu, Gaowei Hu, Siqi Lian, Jiaqi Liu, Shengmei Pang, Guoqiang Zhu, Xueyan Ding

**Affiliations:** 1 College of Veterinary Medicine, Henan Agricultural University, Zhengzhou 450046, China; 2 Molecular Biology Laboratory, Zhengzhou Normal University, Zhengzhou 450044, China; 3 College of Life Sciences, Taizhou University, Taizhou 318000, China; 4 College of Veterinary Medicine, Yangzhou University, Yangzhou 225009, China; 5 Henan Province Key Laboratory of Animal Food Pathogens Surveillance, Zhengzhou 450046, China; 6 Ministry of Education Key Laboratory for Animal Pathogens and Biosafety, Zhengzhou 450046, China

**Keywords:** antimicrobial resistance, antibiotic overuse, resistance mechanisms, countermeasure strategies, antimicrobial development

## Abstract

The discovery and clinical deployment of antibiotics marked a paradigm shift in combating bacterial infections, profoundly benefiting human medicine, veterinary health, and agricultural productivity. However, pervasive overuse in both the clinical and livestock sectors has precipitated an alarming acceleration of antimicrobial resistance, now recognized as a critical global health threat. Compounding this crisis, livestock-associated resistant pathogens persistently colonize the food production continuum, enabling zoonotic transmission through animal-derived products and endangering public health, food safety, and human survival. This review delineates current insights into bacterial resistance mechanisms and evaluates innovative countermeasures, aiming to inform future antimicrobial development and resistance containment strategies.

## 1. Introduction

Antibiotic drugs are metabolic products synthesized by microorganisms (including bacteria, fungi, and actinomycetes) or plants and animals during their growth and development. These products possess antimicrobial activity and can interfere with the chemical substances required for cell development, thus having the ability to kill or inhibit bacteria. They are one of the most commonly used drugs in clinical practice and can play a role in preventing and treating bacterial infectious diseases. However, with the misuse of antibiotics in both human and livestock farming, the situation of bacterial resistance has been continuously worsening, and bacterial antibiotic resistance has become an increasingly serious public health problem [1].

Antimicrobial resistance (AMR) refers to the tolerance of microorganisms, especially bacteria, to antibiotic drugs. This tolerance makes diseases that could originally be treated with antibiotics difficult to cure. AMR can be formed through genetic mutations in bacteria or by acquiring foreign resistance genes. These resistance genes encode enzymes or membrane proteins that can reduce the entry of antibiotics into the cell or degrade antibiotics, allowing bacteria to survive and continue to reproduce in the presence of antibiotics [2]. The accumulation of resistant bacteria in animals not only increases the difficulty of treating animal diseases, but more importantly, they can be transmitted to humans through the food chain, causing human infections that are often difficult to cure with traditional antibiotics [3]. Once resistance genes are exchanged between human and animal pathogens, it will greatly increase the complexity and cost of public health prevention and control [4].

Therefore, it is imperative to accelerate the development of novel antibiotics and innovative antimicrobial strategies to address the escalating public health challenge posed by globally prevalent antibiotic-resistant infections, thereby ensuring the efficacy and sustainability of infectious disease treatment. Against this backdrop, this review elucidates bacterial resistance mechanisms from three key perspectives: intrinsic resistance, acquired resistance, and adaptive resistance. It also summarizes therapeutic strategies to combat resistant strains including the utilization of traditional Chinese medicine (TCM)-based antimicrobial adjuvants, the targeted disruption of quorum sensing system (QSS), the antibacterial strategies based on the mutant selection window (MSW), and the CRISPR-Cas system’s precision elimination capabilities. Furthermore, by critically comparing the advantages and limitations of diverse antimicrobial approaches, we evaluate their clinical translation potential. These efforts aim to provide robust theoretical foundations for mitigating and controlling bacterial antibiotic resistance.

## 2. Resistance Mechanisms of Bacteria

The evolution and spread of bacterial antibiotic resistance are closely related to human activities, particularly the overuse and misuse of antibiotics. In the medical field, the irrational use of antibiotics includes not only an over-reliance on the treatment of infectious diseases, but also the improper use of antibiotics such as insufficient dosage or too short a course of treatment, which may promote the emergence of drug-resistant strains [5]. In agriculture and animal husbandry, antibiotics are widely used as growth promoters or to prevent infections, which also accelerates the spread of drug-resistance genes [6,7,8]. Additionally, environmental factors, such as antibiotic residues and drug-resistance genes in water and soil, have become a vehicle for the spread of drug-resistant bacteria, posing a potential threat to human health. Bacteria have many causes and complex mechanisms of drug-resistance, which are mainly divided into intrinsic drug-resistance, acquired drug-resistance, and adaptive drug-resistance [9].

### 2.1. Intrinsic Resistance

Intrinsic resistance is an innate characteristic of bacteria, which are naturally resistant to certain antibiotics. This type of resistance is not acquired but determined by the bacterial genome, with the genes encoding resistance located on the bacterial chromosome and being relatively stable [10]. Certain bacterial genera or species possess unique structural features and functions that make them naturally insensitive to drugs. These bacteria typically lack the targets for antibiotics, making them resistant to the bactericidal or inhibitory effects of these drugs [11].

A typical example is the intrinsic resistance of Gram-negative bacteria to certain bactericidal agents and antibiotics, which is mainly attributed to their unique cell structures and metabolic differences. Taking the bactericidal agent triclosan as an example, it can effectively inhibit Gram-positive bacteria and many Gram-negative bacteria, but it has little effect on *Pseudomonas*, a genus of Gram-negative bacteria. This is because the mechanism of action of triclosan involves targeting the enzyme enoyl-ACP reductase in bacteria. However, in *Pseudomonas*, there is an insensitive allele (such as *fabI*) that prevents triclosan from effectively acting on the enzyme, thus conferring intrinsic resistance to triclosan [12]. Similarly, the lipopeptide drug daptomycin shows good antimicrobial effects against Gram-positive bacteria but is less effective against Gram-negative bacteria. The reason behind this lies in the significant differences in cell membrane structures between Gram-negative and Gram-positive bacteria. Specifically, the proportion of anionic phospholipids in the cytoplasmic membrane of Gram-negative bacteria is relatively low, which affects the efficiency of Ca^2+^-mediated daptomycin entry into the cell membrane [13]. Moreover, the intrinsic resistance of Gram-negative bacteria to a variety of drugs is also related to their outer membrane structure. The outer membrane of Gram-negative bacteria contains a layer of lipopolysaccharides (LPSs) that forms a powerful permeability barrier that prevents the entry of drugs. For instance, the glycopeptide antibiotic vancomycin, which inhibits peptidoglycan cross-linking by binding to the D-alanine-D-alanine dipeptide, cannot penetrate the outer membrane barrier of Gram-negative bacteria and is thus ineffective against them. This phenomenon fully demonstrates the effectiveness of the natural barrier of Gram-negative bacteria and the complexity of intrinsic resistance [14].

### 2.2. Acquired Resistance

**Figure 1:**
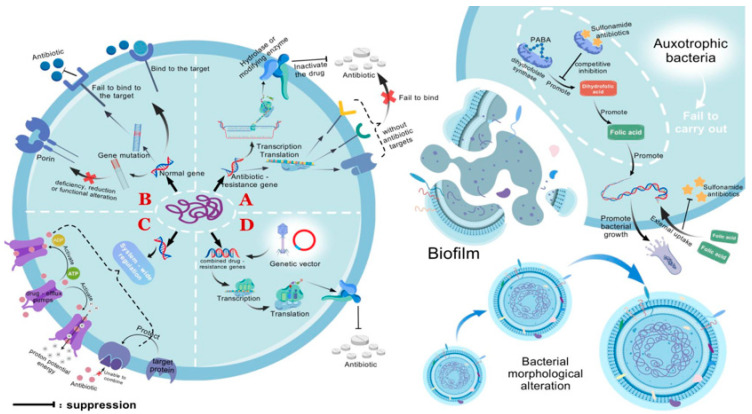
**Bacterial resistance mechanisms.** (A) Bacteria containing antibiotic resistance genes are incapable of producing the drug targets for antibiotics, preventing the antibiotics from binding and exerting their effects (intrinsic resistance). Resistance genes can also encode enzymes that hydrolyze antibiotics, rendering them inactive. (B) Genetic mutations can alter the drug targets in bacteria, preventing antibiotics from binding to the targets as usual. Alternatively, they can change the number or function of porins, reducing the permeability of the cell membrane and thereby decreasing drug penetration. (C) Through genetic regulatory systems, drug efflux pumps can be activated to lower the concentration of drugs within the cell. Additionally, target-protecting proteins can be produced to prevent the binding of targets to drugs. (D) Resistance genes can be disseminated to susceptible bacteria via genetic vectors such as plasmids and bacteriophages, conferring resistance upon them. In addition, adaptive resistance includes the formation of biofilms, changes in metabolic pathways and nutritional deficiencies, and alterations in cell morphology. Nutritional deficient bacteria are unable to synthesize folic acid on their own and must acquire it from the external environment, thereby inhibiting the action pathway of sulfonamide antibiotics and promoting bacterial growth. PABA, p-aminobenzoic acid. This figure was created by BioGDP.com [17].

Acquired resistance refers to the phenomenon where bacteria that were originally sensitive to a particular antibiotic become resistant to it after exposure to the antibiotic. This resistance is achieved through genetic mutations or the acquisition of exogenous resistance genes, which alter the bacteria’s metabolic pathways and enable them to avoid being inhibited or killed by the drug [15,16]. This type of resistance is acquired and differs from the bacteria’s original genome, typically occurring under the selective pressure of antibiotics (Figure 1).

#### 2.2.1. Changing the Drug Targets by Genetic Mutation or Modification

Bacteria can alter the structure of antibiotic targets to reduce the affinity between antibiotics and their targets, thereby inducing resistance. Target alterations are typically caused by spontaneous mutations in bacterial genes on the chromosome. Since the interaction between antibiotics and target molecules is usually quite specific, even minor changes in the target molecule can significantly impact antibiotic binding [10].

Among the various mechanisms by which bacteria develop resistance through target site mutations, genetic alterations in the quinolone resistance-determining regions (QRDRs) represent the most characteristic paradigm. If envisioning quinolone molecules as keys, the active pockets of DNA gyrase and topoisomerase IV would constitute the corresponding keyholes. Structurally, DNA gyrase is primarily assembled through the GyrA and GyrB subunits, while topoisomerase IV relies on the coordinated action of ParC and ParE subunits [18,19]. As early as the 1990s, electrophoretic-sequencing experiments revealed that the shape of these keyholes could be perturbed in four specific amino acid segments—GyrA, GyrB, ParC, and ParE—analogous to arbitrary keyhole modifications. Such alterations create structural incompatibilities that prevent proper quinolone binding, akin to a misfit key failing to engage with a reshaped lock [18].

Taking *Escherichia coli* (*E. coli*) as a prototypical example, the QRDRs within the DNA gyrase-encoding gene encompass critical amino acid segments: positions 67–106 of the GyrA subunit and positions 426–447 of the GyrB subunit. Similarly, in the topoisomerase IV encoding system, the ParC subunit’s residues 63–102 and the ParE subunit’s residues 420–441 constitute equally essential QRDR components [20,21]. Any single amino acid substitution within these functional domains can compromise the molecular binding affinity between target enzymes and quinolone antibiotics. This reduced binding capacity directly diminishes the drugs’ antimicrobial efficacy, thereby facilitating bacterial resistance development. Notably, among clinically isolated resistant strains, the Ser83Leu/Ile substitution in the GyrA subunit represents the most prevalent resistance genotype, where this specific amino acid alteration significantly weakens DNA gyrase–quinolone interactions [22].

The failure of penicillin-class antibiotics often originates from the bacteria’s subtle genetic modifications. β-Lactam drugs typically target penicillin-binding proteins (PBPs) located on the cytoplasmic membrane, which play a crucial role in synthesizing peptidoglycan—an essential component for bacterial growth and division [23]. When penicillin binds to PBPs, cell wall synthesis is inhibited, leading to bacterial lysis. However, antibiotic misuse has driven bacteria to evolve multiple mechanisms that alter PBP structures, thereby reducing the affinity between β-lactams and PBPs. This structural adaptation prevents effective antibiotic binding, ultimately rendering the drugs ineffective [24]. A classic example is *Staphylococcus aureus* (*S. aureus*) acquiring the *mecA* gene, which encodes a modified PBP variant (PBP2a) with significantly reduced β-lactam binding affinity. This structural change enables bacteria to maintain normal peptidoglycan synthesis and cell wall integrity even in β-lactam-rich environments, conferring resistance [25,26]. Compounding this challenge is the natural variability among bacterial strains in PBP types and quantities, which provides diverse resistance strategies. Beyond PBP2a-mediated resistance, bacteria may employ alternative approaches: upregulating PBP expression, modifying active sites, or introducing additional PBP variants. These mechanisms collectively enhance bacterial resilience against β-lactam antibiotics, creating a complex clinical challenge [27].

In addition to direct gene mutations, bacteria can also develop resistance to a variety of antibiotics through chemical modifications of gene products by specific enzymes, a mechanism that is particularly significant in AMR research. Methylation modification of 23S rRNA is a notable way for bacteria to develop drug resistance. This modification is catalyzed by specific enzymes, and the gene expression of these enzymes is directly related to the cross-resistance of bacteria to antibiotics. The enzyme encoded by the *erm* (erythromycin ribosomal methylase) gene can methylate specific regions of bacterial 23S rRNA. This chemical modification alters the structure of the ribosome, reducing the binding ability of macrolides, lincosamides, and streptogramin B antibiotics to the ribosome [28], thereby conferring cross-resistance to these antibiotics on the bacteria. The enzyme encoded by the *cfr* (chloramphenicol-florfenicol resistance) gene can produce a different methylation modification on 23S rRNA, enabling bacteria to develop resistance to linezolid, chloramphenicol, and clindamycin. Linezolid is a synthetic oxazolidinone antibiotic, while chloramphenicol and clindamycin belong to the chloramphenicol and lincosamide classes of antibiotics, respectively. Although these antibiotics have different mechanisms of action, their resistance mechanisms can be achieved through the methylation modification of 23S rRNA mediated by the enzyme expressed by the *cfr* gene [29].

#### 2.2.2. Reduced Drug Concentration Caused by Changing the Permeability of the Cell Membrane

The efficacy of antibiotics in combating bacterial infections depends critically on their ability to traverse the bacterial cell wall and membrane systems to reach intracellular targets. The structural dichotomy between Gram-negative and Gram-positive bacteria profoundly influences antibiotic susceptibility. Gram-negative bacteria possess a dual-membrane architecture, with an outer membrane enriched in LPS and selective porins (i.e., transmembrane proteins) that mediate solute exchange while simultaneously acting as permeability barriers. In contrast, Gram-positive bacteria lack this outer membrane, exhibiting a simpler cell wall structure that facilitates antibiotic penetration [30,31,32]. Porins, particularly non-specific channels, such as outer membrane protein (Omp)F and OmpC in Enterobacteriaceae, enable the passive diffusion of hydrophilic antibiotics including β-lactams, fluoroquinolones, tetracyclines, and chloramphenicol across the outer membrane [33,34]. Earlier studies posited selective porin–antibiotic interactions, however, recent investigations have revealed that antibiotic permeation predominantly occurs via stochastic passive diffusion processes [35]. Bacteria can reduce the permeability of their outer membrane by adjusting the quantity or pore size of porins as well as altering the thickness of their cell wall, thereby decreasing the efficiency with which antibiotics reach their targets. Bacterial limitation of antibiotic uptake typically involves two mechanisms: the absence or significant reduction in porins, and alterations in porin function or selectivity due to mutations [36].

Genetic mutations affecting porins can manifest as complete porin loss, reduced expression, or structural distortions, all of which contribute to antibiotic resistance. For instance, prolonged antibiotic exposure in clinical settings often selects for mutations in the *ompF* gene, leading to diminished OmpF channel density in Gram-negative pathogens. This adaptive response reduces the intracellular accumulation of β-lactams and fluoroquinolones, synergizing with other resistance mechanisms such as efflux pump hyperactivity or enzymatic drug inactivation to confer high-level resistance [37,38]. Porin expression is tightly regulated by sophisticated genetic circuits. In *E. coli*, the EnvZ-OmpR two-component regulatory system modulates the reciprocal expression of OmpC and OmpF in response to osmotic stress. EnvZ, a periplasmic histidine kinase, phosphorylates the transcription factor OmpR, which subsequently activates *ompC* transcription while repressing *ompF*. This dynamic regulation fine-tunes membrane permeability and antibiotic susceptibility [39]. Such regulatory pathways have emerged as novel targets for antimicrobial development. Notably, *Mycobacterium tuberculosis* (*M. tuberculosis*), though lacking canonical porins, employs the PPE51 protein to facilitate solute transport. The small-molecule inhibitor 3bMP1, which targets PPE51, has demonstrated potent anti-tubercular activity in vitro, highlighting the therapeutic potential of porin-like structure modulation [40].

#### 2.2.3. Drug Inactivation Due to Synthetic Hydrolase or Modified Enzyme

Bacterial resistance to antibiotics is a complex and multifaceted issue, and the inactivation or modification of antibiotics is a common mechanism by which bacteria develop resistance. Unlike other resistance mechanisms that rely on altering cellular functions to counteract the effects of antibiotics, this mechanism uniquely acts directly on the antibiotic molecules themselves, without affecting the normal physiological processes of the bacteria. Bacteria can produce specific enzymes, such as hydrolases or inactivating enzymes, that directly interfere with the action of antibiotics [41]. These enzymes are capable of breaking down or chemically altering antibiotic molecules, rendering them inactive before they reach the bacterial target sites and thus unable to exert their antimicrobial effects. The modification mechanisms of antibiotics can be divided into two categories: one is the direct degradation of antibiotics, where enzymes catalyze reactions to destroy the structure of antibiotics, causing them to lose their antimicrobial activity; the other is through the transfer of chemical groups, where enzymes transfer specific chemical groups from antibiotic molecules to other molecules, also achieving the purpose of inactivating the antibiotics [10].

Bacteria can produce specific inactivating or detoxifying enzymes. These enzymes include, but are not limited to, beta-lactamases, chloramphenicol acetyltransferase (CAT), aminoglycoside inactivating enzymes, and macrolide-lincosamide-streptogramin inactivating enzymes, each targeting different categories of antibiotics and exhibiting different inactivation pathways. Beta-lactamases are enzymes encoded by bacteria themselves, specifically targeting beta-lactam antibiotics such as penicillin and cephalosporin. These enzymes covalently bind to the beta-lactam ring in the antibiotic molecule, destroying its structure and causing the antibiotic to be degraded before it reaches the bacterial target [42,43]. In addition, beta-lactamases can also bind to antibiotics in a non-hydrolytic manner, preventing their interaction with the target site and thus conferring resistance on the bacteria. *E. coli*, *Pseudomonas aeruginosa* (*P. aeruginosa*), and *Acinetobacter baumannii* (*A. baumannii*), among other bacteria, can express a flavin-dependent monooxygenase Tet(X) that inactivates tigecycline and other tetracycline antibiotics by degrading them and destroying their antimicrobial activity [44,45]. Some Microbacterium species, on the other hand, cause structural changes in sulfonamide antibiotics through the combined action of sulfonamide monooxygenase (SadA) and flavin reductase (SadC), rendering them ineffective [46]. Enterobacteriaceae bacteria effectively disrupt the structure of macrolide antibiotics and weaken their antimicrobial effects by producing glycosidases, esterases, and phosphatases [47]. For aminoglycoside antibiotics, inactivating enzymes such as N-acetyltransferase, O-phosphotransferase, and O-adenylyltransferase can acetylate, phosphorylate, or adenylylate these antibiotics, altering their structure through chemical modification and causing them to lose their antimicrobial activity [6].

Bacteria can produce enzymes that link a variety of active groups (such as acetyl, phosphoryl, and adenosyl groups) to antibiotics, altering their original chemical properties and preventing antibiotics from binding to their target sites, thus rendering them ineffective against bacteria. For example, aminoglycoside-modifying enzymes, including phosphotransferases, acetyltransferases, and nucleotidyltransferases, can covalently modify the amino or hydroxyl groups on aminoglycoside antibiotic molecules, preventing the antibiotics from binding to their bacterial target sites (usually the ribosome) and thus conferring resistance [48]. This mechanism, by altering the structure of the antibiotic, prevents the antibiotic from inhibiting bacterial protein synthesis. CAT, on the other hand, catalyzes an acetyl-CoA-dependent reaction that acetylates the hydroxyl group at position C in chloramphenicol molecules, forming 1-acetylchloramphenicol. This chemical modification prevents the antibiotic from binding to the bacterial ribosome, thereby conferring resistance to chloramphenicol on the bacteria. The ADP-ribosyltransferase in *Mycobacterium abscessus* can catalyze the connection of ADP-ribose to the hydroxyl group at position C23 of rifampicin, thereby blocking its binding to RNA polymerase, which is a key mechanism leading to rifampicin resistance [49]. Glycosyltransferases, with functions similar to ADP-ribosyltransferases, glycosylate the hydroxyl group at position C23 of rifampicin, similarly blocking the activity of the antibiotic [50]. Phosphotransferases act on position C21 of rifampicin, converting it into inactive phosphorylated rifampicin, and the naphthyl part of rifampicin is also oxidized by monooxygenases, losing its ability to bind to the RNA polymerase beta subunit (RpoB) protein, thus failing to inhibit bacterial RNA synthesis [51,52].

#### 2.2.4. The Reduction in Drug Accumulation Caused by the Activation of the Drug Efflux Pump

The diversity and complexity of resistance mechanisms in bacteria are exemplified by efflux reactions, which serve as an immediate and efficient strategy for bacteria to counteract antibiotic stress [53]. Bacteria utilize efflux pump systems, protein complexes embedded in the cell membrane, to non-specifically pump antibiotics and other drugs out of the cell. This process maintains intracellular drug concentrations below the minimum inhibitory concentration (MIC), thereby avoiding being killed by antibiotics and exhibiting drug tolerance. Different efflux pumps vary in their degree of response to and selectivity for drugs, with some conferring high levels of resistance and others providing only low levels of protection [54]. The differences in selectivity and response highlight the fine-tuned regulation of efflux pump systems in the evolution of bacterial resistance. Efflux systems consist of three key components: efflux proteins, fusion proteins, and outer membrane channel proteins, which work together to ensure the effective expulsion of drugs. Based on structural characteristics and energy supply methods, bacterial efflux system transport proteins can be classified into five major families: the resistance-nodulation-division family (RND), the ATP-binding cassette family (ABC), the major facilitator superfamily (MFS), the small multidrug resistance family (SMR), and the multidrug and toxic compound extrusion family (MATE) [55,56]. The first type is powered by ATP hydrolysis, while the latter four use the electrochemical proton gradient to expel drugs out of the cell.

In Gram-positive bacteria, efflux pump systems mainly include four categories: MFS, SMR, MATE, and ABC, with MFS family members being renowned for their specific efflux pump functions [10,57]. *Streptococcus pneumoniae*, a typical representative of Gram-positive bacteria, carries not only MFS-type efflux pumps (such as the Mef pump), but also ABC-type efflux pumps (such as PatA and PatB pumps). The Mef pump is particularly noteworthy because it is specifically responsible for the efflux of macrolide antibiotics. Macrolides inhibit protein synthesis by binding to bacterial ribosomes and forming macrolide–ribosome–mRNA complexes. The transcriptional regulation mechanism of the Mef efflux pump is in line with this mode of action [57], ensuring that its expression gene is not suppressed, allowing the pump protein to be fully expressed and effectively expel the drug out of the cell [10].

In Gram-negative bacteria, efflux pumps work in conjunction with the unique structure of the double-layered cell membrane, endowing these bacteria with resistance to a wide range of antibiotics. RND family efflux pumps are known for their multi-component structure and synergistic mechanism, often working with periplasmic membrane fusion proteins and OMPs to efficiently expel substrates into the extracellular environment. RND efflux pumps are closely related to bacterial resistance capabilities due to their broad substrate range including structurally diverse antibiotics [58]. For example, the key efflux pump AcrAB-ToIC (a tripartite complex comprising the AcrB inner membrane transporter, the AcrA membrane fusion protein, and the ToIC outer membrane channel) in *E. coli*, a member of the RND family, has its expression of the AcrAB efflux pump inhibited by the regulatory protein AcrR in the absence of drug stimulation. However, when the cell is exposed to drugs, the AcrR protein can bind to the drug, causing a conformational change that relieves the inhibition of AcrAB and activates the drug efflux function of the pump. In *M. tuberculosis*, several efflux pump genes associated with drug tolerance have also been reported. For instance, the EfpA protein of the MFS family is related to resistance to isoniazid, fluoroquinolones, rifampicin, and clofazimine [59]; the Mmr protein of the SMR family is associated with resistance to ofloxacin and rifampicin [60]; and the MmpS5–Mmpl5 complex of the RND family is related to the level of bedaquiline resistance [60].

#### 2.2.5. The Production of Target Protective Proteins with Inhibitory Drug Effects

Bacteria can synthesize specific proteins that effectively prevent antibiotics from binding to their targets, thereby eliminating the antimicrobial effects of antibiotics. This mechanism is evident in both Gram-negative and Gram-positive bacteria. Ribosomal protection proteins are a key category of defense molecules that protect the ribosome from antibiotic inhibition by binding to the antibiotic target site or altering the structure of the ribosome, thereby conferring antibiotic resistance on the bacteria [61,62]. For example, the Qnr protein can mimic the action of DNA to prevent the interaction between gyrase and topoisomerase I and bacterial DNA, reducing the binding sites for quinolone antibiotics within the bacteria and thus mediating the emergence of quinolone resistance [63].

According to their mechanisms of action, target protection proteins can be broadly classified into three categories [64]. (1) Type I target protection proteins: These proteins physically block the binding of antibiotics to their targets by directly covering the antibiotic binding sites. For instance, tetracycline ribosome protection proteins (TRPPs) can bind to the ribosome, altering its structure and thus interfering with the binding of tetracycline to 16S rRNA. This causes tetracycline drugs to dissociate from the ribosomal binding site, protecting the ribosome from the effects of antibiotics [65,66]. (2) Type II target protection proteins: These proteins induce the dissociation of bound antibiotics by altering the conformation of the target. Their function is typically mediated by antibiotic resistance ABC-F proteins. Taking the VmlR protein (TetR family transcriptional regulator) as an example, the ribosome fixed by antibiotics is recognized by the VmlR protein, which binds to the E site of the ribosome. The C-terminal of the VmlR protein extends into the mRNA export channel, inducing a distortion in the structure of P-tRNA at the P site. This prompts the antibiotic resistance domain of the ABC-F protein to enter the peptidyl transferase center and the adjacent nascent polypeptide export channel, thereby causing the drug to dissociate. After hydrolyzing ATP into ADP, the ABC-F protein transitions into a low-affinity open conformation and dissociates from the ribosome, restoring the bacterial protein translation process [67,68]. (3) Type III target protection proteins: These proteins induce conformational changes in the target, allowing it to function normally even when antibiotics are bound. For example, the FusB protein can bind to elongation factor G and drive its dissociation from the ribosome, thereby conferring resistance to fusidic acid in *S. aureus* [69,70].

#### 2.2.6. Resistance Genes Obtained by Gene Transfer

In the long-term evolution of bacteria, horizontal gene transfer (HGT) is a crucial genetic mechanism that can transfer resistance genes into susceptible bacteria. These resistance genes produce a range of weapons to combat antibiotics including enzymes that destroy antibiotics, drug efflux pumps, and the loss or narrowing of cell membrane porins [71]. HGT greatly promotes gene exchange between bacterial populations, especially the spread of resistance genes, significantly enhancing the survival adaptability of bacteria. Its modes of occurrence include conjugation, transformation, and transduction [72]. This process often relies on the action of mobile genetic elements, which can move between different DNA molecules, including insertion sequences (IS), transposons, integrases, and plasmids that can cross bacterial cell boundaries, constituting the main vehicles for the spread of resistance genes.

Plasmids, as important mobile genetic carriers, vary widely in size, ranging from thousands to millions of base pairs. They intercept and carry antibiotic resistance genes between bacteria, which are usually connected to one or more resistance-related genes and mobile genetic elements [73,74,75,76,77]. The resistance genes on plasmids can not only be vertically transmitted to offspring during cell division, but also, through HGT, cross different bacterial populations to achieve the widespread dissemination of resistance genes [78,79,80]. A typical example is the *mecA* gene, which originally came from coagulase-negative *staphylococci* or *enterococci*. Through plasmid-mediated transfer, it moved to *S. aureus* and further integrated into the host bacterium’s chromosome via transposons, conferring resistance to beta-lactam antibiotics on what was originally a susceptible *S. aureus* [81].

Bacteriophages, as a class of viruses that infect bacteria, transfer DNA fragments from one bacterium to another through genetic transduction, becoming an important driving force in microbial evolution and the spread of resistance genes [82,83,84]. Known transduction mechanisms include generalized transduction (GT) and specialized transduction (ST). GT refers to the process in which bacteriophages randomly package host DNA fragments and transfer them to another bacterium. In this process, bacteriophages may spontaneously lyse or be induced to lyse by bacterial stress responses, releasing transducing particles carrying host DNA and transferring the DNA to new host cells. ST, on the other hand, is limited to the transfer of specific genomic fragments [6,85,86]. One study revealed a new mechanism called lateral transduction, which can transfer large segments of the bacterial chromosome from one bacterium directly to another with extremely high efficiency [87]. In addition, some bacteriophages can enhance bacterial resistance by forming physical barriers around host bacteria such as the liquid crystal structures built by filamentous bacteriophages, which isolate antibiotics [88].

### 2.3. Adaptive Resistance

Adaptive resistance is a dynamic and reversible phenomenon of antibiotic resistance in bacteria in response to specific environmental signals, allowing them to temporarily withstand the bactericidal effects of one or more antibiotics. The triggers for this resistance are diverse, including but not limited to external stress, bacterial growth status, environmental pH, ion concentration, nutritional conditions, and low-concentration (sub-inhibitory level) exposure to antibiotics [89,90,91,92]. Unlike intrinsic and acquired resistance, adaptive resistance is a temporary state, closely related to the presence or absence of environmental signals. As an epigenetic response to environmental adaptation, adaptive resistance does not involve changes in DNA sequences but is achieved through the regulation of gene expression. The formation of this resistance enables bacteria to rapidly adjust their survival strategies under antibiotic pressure, and once the pressure is removed, the bacteria’s sensitivity will also be restored [93].

Under the long-term effect of sub-inhibitory-level antibiotics, bacterial populations will undergo a series of adaptive evolutionary processes. Initially, bacteria may only resist antibiotics through adaptive resistance mechanisms, which are effective but unstable. However, with the continuous action of antibiotics, adaptive evolution may further drive the development of more effective and lasting resistance mechanisms in bacteria [94]. For example, bacteria may enhance the activity of drug efflux pumps through gene mutations, or alter the permeability of the cell membrane, or even acquire new resistance genes. Cui et al. described “genome flip-flop inversion” as a reversible bacterial survival mechanism involving large-scale chromosomal inversions, whereby large chromosomal segments undergo reversible flipping to dynamically alter the expression of genes within or adjacent to the inverted regions [95].This structural plasticity directly modulates key phenotypes, most notably antibiotic susceptibility, enabling bacteria to switch between susceptible and resistant states in response to environmental pressures such as antibiotic exposure. As a critical component of adaptive resistance strategies, this reversible inversion allows bacteria to rapidly adjust their fitness without accumulating permanent genetic mutations, providing an energy-efficient and flexible means to survive fluctuating hostile environments.

This evolution from adaptive resistance to stable resistance reveals the complexity and diversity of bacterial survival strategies under antibiotic selective pressure [96]. Therefore, adaptive resistance is not only a strategy for bacteria to respond to antibiotics in the short-term, but also a crucial link in their long-term evolution of resistance (Figure 1).

#### 2.3.1. Changes in Metabolic Pathways and Nutritional Deficiencies

In the metabolic networks of bacteria, mutations in specific genes can significantly impact their sensitivity to antibiotics, revealing the subtle but crucial link between metabolic pathways and drug resistance. In 2021, a groundbreaking study by Allison et al. first revealed that mutations in core genes of metabolic pathways could directly or indirectly trigger AMR [97]. Taking the 2-ketoglutarate dehydrogenase gene as an example, this gene plays a key role in the tricarboxylic acid (TCA) cycle, catalyzing the conversion of 2-ketoglutarate to succinyl-CoA. When this gene mutates, the activity of the TCA cycle is inhibited, and the respiration of bacteria is weakened, thus avoiding the toxicity caused by excessive metabolic activity, and ultimately leading to the increased tolerance of bacteria to certain antibiotics [66].

These findings not only deepen our understanding of the relationship between bacterial metabolism and antibiotic sensitivity, but also propose a new perspective: alterations in metabolic pathways are one of the key drivers of AMR. For instance, auxotrophic strains, which lack the ability to synthesize essential nutrients, such as amino acids, nucleotides, vitamins, fatty acids, or metabolic cofactors, rely on the uptake of these metabolites from the environment to sustain survival [98,99,100,101]. This dependency, under the influence of antibiotics, can instead provide a potential survival advantage for bacteria. Sulfonamides, as classic antibiotics, function by mimicking para-aminobenzoic acid and competitively binding to the active site of dihydrofolate synthase, thereby inhibiting the synthesis of dihydrofolate, blocking the folate metabolic pathway, and subsequently affecting nucleic acid synthesis to inhibit bacterial growth. However, for auxotrophic bacteria that can directly obtain folate from the external environment, the efficacy of this mechanism is significantly reduced because they can bypass the blockage by sulfonamides and maintain normal folate metabolism and growth. Auxotrophic bacteria, due to the lack of certain essential nutrients, adapt to the environment by adjusting their metabolic pathways. This metabolic adaptability may enable them to survive under the presence of antibiotics, thereby exhibiting enhanced tolerance [102]. In addition, some bacteria enter a “persister” state in nutrient-depleted environments, and bacteria in this state show significantly increased tolerance to antibiotics [103]. It can be inferred that there may be a close link between the metabolic adaptability of auxotrophic bacteria and antibiotic tolerance. Under specific conditions, the auxotrophic state not only does not weaken the survival ability of bacteria, but can also enhance their tolerance to antibiotics [104,105]. This phenomenon suggests that by modulating the nutritional status of bacteria, it may be possible to develop new strategies to overcome AMR. Therefore, limiting the availability of specific nutrients in the environment may inhibit the growth of drug-resistant bacteria or enhance the efficacy of existing antibiotics.

#### 2.3.2. Changes in Cell Morphology

The dynamic changes in cell morphology significantly impact the inhibitory effects of antibiotics, a phenomenon closely related to the growth state and morphological characteristics of bacteria. When bacteria increase in size, it is equivalent to increasing the internal volume, which helps dilute the concentration of antibiotics entering the cell, thereby reducing the bactericidal power of antibiotics [106,107,108]. In addition, bacteria can effectively reduce the surface-to-volume ratio (specific surface area) by changing their shape, such as becoming more curved or increasing the surface area, thereby reducing the accumulation of antibiotics on the cell surface. This physical mechanism enables bacteria to more effectively resist the attack of antibiotics [106,107,109].

The morphological transformation of *Caulobacter crescentus* (*C. crescentus*) observed by Ojkic et al. under antibiotic stress is particularly striking [106]. Under the pressure of antibiotics, the cell wall of *C. crescentus* undergoes severe stretching, forming a unique “C”-shaped structure. This morphological change not only helps the bacteria reduce the accumulation of antibiotics on the surface, but also unexpectedly promotes the recovery of their growth rate, enabling them to quickly return to a normal growth state. Once the antibiotics are removed, *C. crescentus* can gradually revert to its original shape within a few generations, demonstrating high reversibility and adaptability. This strategy of enhancing antibiotic tolerance through morphological changes is essentially a self-protection mechanism of bacteria. It not only reduces the effective action time of antibiotics on the bacterial surface, but also increases the concentration threshold of antibiotics that bacteria can tolerate [110].

#### 2.3.3. Formation of Biofilms

Antibiotic tolerance in biofilm communities exhibits a twofold enhancement, a phenomenon not attributable solely to physical barrier effects but rather resulting from complex interactions between metabolic, population dynamics, and other multifactorial mechanisms. Biofilms themselves are intricate networks composed of bacterial-secreted polysaccharides, fibrin, and lipoproteins, encasing microbial communities within dense membranous aggregates [111]. As protective structures formed by bacterial populations in specific environments, their primary mechanisms manifest through: (1) restricted antibiotic penetration—the dense architecture of biofilms acts as a physical barrier, impeding antibiotic diffusion into bacterial cells and directly diminishing bactericidal efficacy; (2) metabolic slowdown and growth arrest—biofilm-residing bacteria frequently enter metabolically reduced and growth-arrested states, while antibiotics typically target actively dividing cells—this ‘low-activity’ condition significantly reduces bacterial susceptibility to antimicrobial agents; (3) physiologically reinforced resistance—under antibiotic stress, biofilm bacteria activate stress responses including modulating membrane permeability to limit drug entry or enhancing efflux systems to expel intracellular antibiotics, thereby preventing cellular damage [112].

Emerging evidence demonstrates that biofilm-embedded bacteria exhibit significantly enhanced antibiotic tolerance compared with their planktonic counterparts, with the MIC reaching 1000–2000 times higher levels [113]. This resistance phenotype is particularly pronounced in polymicrobial biofilms, where interspecies interactions play a pivotal role. A paradigmatic example involves *P. aeruginosa* and *S. aureus*: *P. aeruginosa* induces metabolic reprogramming in *S. aureus*, simultaneously suppressing its growth while conferring vancomycin tolerance [114]. Reciprocally, *S. aureus* promotes bacterial aggregation and biofilm structural remodeling, thereby enhancing *P. aeruginosa*’s resistance to tobramycin [115]. These synergistic interactions underscore the complex nature of biofilm-mediated antibiotic resistance, where microbial communities collectively develop defense mechanisms far more sophisticated than those exhibited by individual species.

Early investigations into antibiotic resistance mechanisms within biofilms predominantly attributed the phenomenon to the physical barrier effect of the extracellular matrix, which was thought to impede antibiotic diffusion [116]. However, subsequent studies revealed that antibiotics such as vancomycin, ciprofloxacin, and levofloxacin exhibited unimpeded penetration through biofilms formed by *S. aureus*, *P. aeruginosa*, and *Klebsiella pneumoniae* [117,118]. This observation prompted researchers to re-examine the role of biofilm microenvironment in antibiotic resistance. Critical findings emerged highlighting that hypoxic and nutrient-deprived conditions within biofilms promote the formation of persister cells—a subpopulation exhibiting extraordinary antibiotic tolerance capable of surviving even in antibiotic-rich environments [119,120]. These discoveries collectively demonstrate that biofilms confer resistance through a dual mechanism: their complex architecture may physically restrict certain antibiotics while simultaneously inducing physiological adaptations in bacteria including metabolic suppression and persister cell formation, thereby significantly enhancing bacterial resilience to antimicrobial agents.

## 3. Strategies to Combat Bacterial Resistance

**Figure 2:**
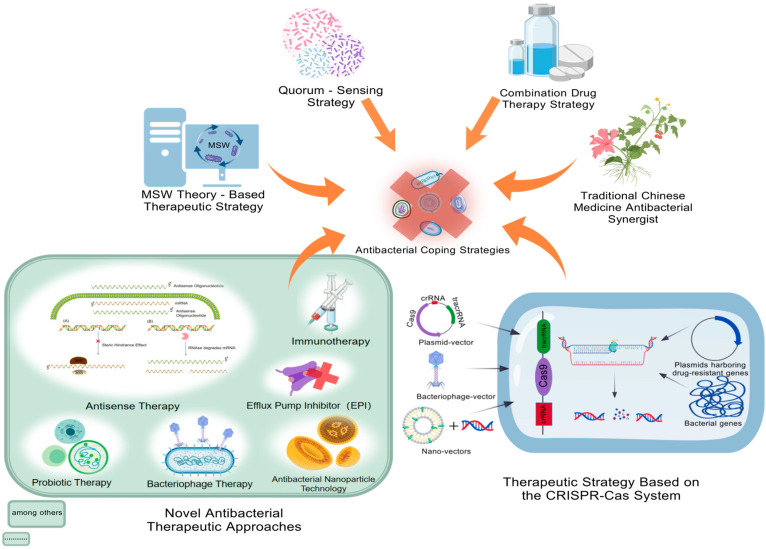
**Antibacterial coping strategies.** Antibacterial coping strategies include the development strategies of TCM antibacterial synergists, QSS strategies, treatment strategies based on the mutant selection concentration of bacterial resistance (MPC, MSW theory), and novel antibacterial treatment methods. Among them, the novel antibacterial treatment methods cover phage therapy, antisense therapy, probiotic therapy, immunotherapy, anti-virulence therapy, and antibacterial peptide therapy. MPC, minimum precautionary concentration; MIC, minimum inhibitory concentration; MSW, mutation selection window. This figure was created by BioGDP.com [17].

AMR has become a major global public health challenge, posing a serious threat to the effectiveness of antibiotics and the safety of human and animal life. With the widespread use and misuse of antibiotics, the emergence and spread of drug-resistant bacteria have significantly accelerated, making many common infections difficult to treat. In response to this increasingly severe challenge, scientists are exploring a variety of innovative antibacterial strategies to prevent the further exacerbation of bacterial drug resistance (Figure 2).

### 3.1. Development of Antibacterial Synergistic Agent of TCM

Plant-derived antimicrobial compounds have demonstrated the ability to reach effective antimicrobial concentrations in the systemic circulation, thanks to the diverse derivatives present in plants that can specifically control the development of drug resistance [121]. For instance, eugenol can effectively inhibit the growth of *E. coli* tolerant to polymyxin at concentrations as low as 4–8 μg/mL [122]. However, most plant-derived antimicrobial agents require higher concentrations to exhibit their effects. For example, cinnamaldehyde can inhibit *E. coli* and *S. aureus* only at a concentration of 0.31 mg/mL [123]. Lactone compounds found in Andrographis paniculata and Artemisia species also need to reach certain concentrations to exhibit good antimicrobial activity [124]. In contrast, the MICs of many clinically used antibiotics are usually between 1 and –2 μg/mL. Given that TCM compounds generally require higher concentrations to exert antimicrobial effects, they are often used as antimicrobial potentiators rather than monotherapeutic agents. For example, trimethoprim, as a potentiator of sulfonamides and other Western medicines, significantly enhances the antibacterial effect when combined with TCMs such as dandelion, honeysuckle, or berberine. Additionally, honeysuckle extract, when used in combination with various antibiotics, has a synergistic potentiating effect on extensively drug-resistant Enterobacteriaceae (XDRE) and non-fermenting bacteria. Research results show that honeysuckle extract, when combined with ceftriaxone, exhibits significant antibacterial effects against drug-resistant *E. coli*; when combined with imipenem, it has a synergistic potentiating effect against drug-resistant *P. aeruginosa*; and when combined with gentamicin, it also has a synergistic potentiating effect against drug-resistant *A. baumannii* [125]. Berberine from *Coptis chinensis*, when used in combination with imipenem, ofloxacin, azithromycin, levofloxacin, ciprofloxacin, gentamicin, and other aminoglycoside drugs, has a synergistic potentiating effect on multiple drug-resistant strains and can effectively enhance the sensitivity of drug-resistant bacteria to antibiotics [126]. Epigallocatechin gallate (EGCG) from green tea, as a promising candidate, not only has synergistic effects with antibiotics, but its multiple antimicrobial mechanisms can also help prevent the development of drug resistance, showing extensive clinical application potential [127].

TCM exhibits multiple mechanisms in reversing bacterial drug resistance including eliminating drug-resistant plasmids, inhibiting beta-lactamases, inducing gene mutations, inhibiting efflux pump activity, disrupting biofilms, altering cell membrane permeability, and inhibiting the activity of enzymes related to metabolic activities. Quercetin and baicalin, components of TCM, when used in combination with various antibiotics, both show significant antimicrobial potentiating effects. Quercetin, when used in combination with ampicillin, exhibits partial synergistic effects against methicillin-resistant *S. aureus* (MRSA) and enhances the antibacterial activity of ampicillin against MRSA as an adjuvant. Additionally, quercetin, when used in combination with levofloxacin, ceftriaxone, and gentamicin, shows potential synergistic activity against clinical strains of *P. aeruginosa* with multiple drug resistances [128]. Baicalin, when used in combination with ampicillin and/or gentamicin, exhibits synergistic effects against oral bacteria, and when used in combination with tigecycline, it has a synergistic potentiating effect against drug-resistant *Klebsiella pneumoniae* [129]. Magnolol, a lignin-like compound isolated from Magnolia officinalis or Michelia chapmanii, restores the therapeutic effect of meropenem on New Delhi metallo-beta-lactamase-1 (NDM-1)-producing *E. coli* by inhibiting beta-lactamase activity [130]. The drug resistance of Gram-negative bacteria is often associated with efflux pumps [131]. Carnosic acid, as an efflux pump inhibitor (EPI), can increase the concentration of antibiotics within bacteria and combat drug-resistant *enterococci* and *S. aureus* [132]. Eugenol can effectively inhibit and disrupt the biofilm of *S. aureus*, including MRSA, inhibiting its formation, disrupting intercellular connections, and decomposing formed biofilms [133]. *Coptis chinensis*, when used in combination with vancomycin, can disrupt the cell wall of MRSA and reduce biofilm formation, enhancing the antibacterial effect. It can also reduce the dosage of vancomycin and decrease adverse reactions. Mechanistic studies show that compounds in *Coptis chinensis* can make the bacterial cell membrane more permeable and prevent the function of efflux pumps, thereby increasing the concentration of vancomycin within the cells [134]. Alicin, from garlic, inhibits the activity of microbial RNA synthetase, affects RNA synthesis, inhibits the proliferation of bacteria and fungi, and can even completely kill bacteria [135].

TCM exhibits multiple advantages in inhibiting bacterial drug resistance. With its rich and diverse resources, numerous studies have confirmed the direct antimicrobial activity of TCM components [128,136,137]. Moreover, TCM and its formulas, with their natural properties, show high efficiency, low toxicity and side effects, and the potential to combat drug resistance, providing new perspectives and solutions for modern medicine. Therefore, the development of antimicrobial TCM potentiators can not only innovate antimicrobial therapies, but also promote the development of compound preparations of traditional Chinese and Western medicines, enhancing clinical treatment effects. This not only extends the use cycle of existing antimicrobial drugs and reduces the dependence on chemical drugs, but also helps to reduce drug residues in animal-derived foods, control the spread of drug resistance, and enrich and develop the profound theory of TCM [138,139].

### 3.2. Strategies Developed Based on QSS

QSS serves as a communication system among bacteria, regulating bacterial gene expression in a population density-dependent manner [140]. This system plays a crucial role in the coordinated behavior of bacterial populations. Drug-resistant bacteria utilize QSS to regulate biofilm formation, virulence factor expression, and growth competition, thereby enhancing their survival capabilities within the host. QS signaling molecules (QSSMs) control the expression of cell density-dependent genes, prompting bacteria to exhibit a range of behaviors at the community level such as virulence factor regulation, biofilm construction, cell differentiation, and extracellular polysaccharide production [141].

There is a close and mutually reinforcing relationship between quorum sensing and bacterial biofilms. On the one hand, quorum sensing signals promote biofilm formation, providing bacteria with a microenvironment that offers physical protection and nutrient sharing. On the other hand, the structure of the biofilm serves as an ideal platform for quorum sensing signal transmission, enhancing infectivity and drug resistance [142]. It has been found that QSS can also lead to bacterial drug resistance by regulating the expression of multidrug-resistant (MDR) efflux pumps and modulating the secretion of related proteins in the bacterial cell membrane [143]. Specifically, QS can regulate the expression of genes related to MDR efflux pumps in bacteria. This regulation not only affects the activity of the efflux pumps, but also creates a feedback loop in which changes in the expression of efflux pump genes influence the production of QSSMs [143]. This further activates quorum sensing signals, reduces intracellular antibiotic concentrations, and thus enhances bacterial drug resistance. Additionally, QSS can promote protein secretion to evade the host immune system. Pathogenic bacteria secrete specific proteins through the cell membrane to evade recognition and attack by the host immune system. QSS regulates this process through eight different secretion systems (T1–T7, T9), enabling bacteria to more effectively adhere to, invade host cells, and produce toxic substances, thereby surviving and proliferating within the host [144].

The development of quorum sensing inhibitors (QSIs) to block quorum sensing signals has emerged as an innovative approach for treating biofilm-associated infections. For instance, enzymes with acyl-homoserine lactone (AHL)-degrading activity, such as AHL-lactonases and AHL-acyltransferases, can effectively degrade AHLs and disrupt quorum sensing signal transmission [145]. Penicillin acylase has also demonstrated the ability to degrade AHLs, providing new ideas for drug design [146]. Strains with anti-QS activity screened from coral communities in the South China Sea by Ma et al. [147] as well as the marine extract KS8 discovered by Busetti et al. [148] have both shown potential in inhibiting biofilm formation and enhancing the efficacy of antibiotics. These natural products or their derivatives, when used in combination with antibiotics, significantly improve the clearance efficiency of drug-resistant bacteria, offering new avenues for the treatment of biofilm-associated infections.

### 3.3. Therapeutic Strategies Based on MSW

The minimum precautionary concentration (MPC) is a critical concentration for antimicrobial drugs to prevent the development of bacterial drug resistance. It defines the range of drug concentrations between the MIC and the MPC, known as the MSW, within which the drug can selectively promote the proliferation of drug-resistant strains [149]. The MSW theory emphasizes that when the drug concentration is insufficient to completely inhibit bacterial growth but is above the MIC, it becomes a breeding ground for drug resistance. However, when the drug concentration reaches or exceeds the MPC, since at least two independent mutations are required for bacteria to grow at this concentration, and such double mutations rarely occur naturally, it can effectively suppress the emergence of drug resistance.

In clinical practice, the selectivity index (SI), which is the ratio of the MPC to the MIC, is used to assess the width of the MSW [150]. A larger SI value indicates a wider MSW, making it easier for antimicrobial drugs to select for drug-resistant strains. Therefore, narrowing or eliminating the MSW is a key strategy for preventing bacterial drug resistance in clinical settings [151] Approaches to achieving this goal include shortening the time spent in the MSW, that is, ensuring that the drug rapidly reaches its peak concentration and crosses the MSW after the initial dose, and then maintaining the concentration above the MPC to minimize the duration of selective pressure. Another approach is to narrow the gap between the MIC and the MPC, that is, by reducing the SI value to decrease the drug’s ability to select for drug-resistant strains [152].

Selecting drugs with a narrow MSW and a low MPC, or using combination therapy to reduce the MPC and narrow the MSW, are effective strategies. However, achieving concentrations above the MPC may cause severe side effects, limiting the applicability of this approach. Combining antibiotics with different mechanisms of action requires bacteria to simultaneously undergo two independent mutations to survive, effectively closing the MSW [153]. When the pharmacokinetics of the drugs are similar, adjusting the dosage and administration schedule to maintain each drug near its respective MIC can result in a synergistic effect and optimized therapeutic outcomes. Therefore, antimicrobial strategies guided by the MSW theory emphasize the importance of optimizing drug selection and usage patterns to shorten or eliminate the MSW, thereby reducing the emergence and spread of drug-resistant strains [154,155,156,157,158].

### 3.4. Targeted Elimination of Drug-Resistant Bacteria Using CRISPR-Cas System

Traditional antibiotics often leave sublethal doses in use, providing an environment for bacteria to generate gene mutations or phenotypic changes, which may in turn lead to the emergence of drug-resistant bacteria. Faced with this challenge, the development of tools that can precisely target and eliminate specific drug-resistant bacteria has become particularly important. The CRISPR-Cas system, with its ability to precisely recognize and degrade specific DNA or RNA sequences, offers an innovative solution to this dilemma. The CRISPR-Cas system consists of CRISPR arrays and genes encoding Cas proteins, and this system has shown great potential in the field of gene editing [159]. By precisely designing single guide RNA (sgRNA) that matches the conserved sequences of drug-resistance genes, the CRISPR-Cas system can target and cleave these drug-resistance genes, leading to their inactivation or loss. When the CRISPR-Cas system is introduced into target drug-resistant bacteria, the Cas protein, guided by sgRNA, precisely cuts the target drug-resistance gene sequences. In the absence of a repair template, bacteria are unable to effectively repair the double-strand DNA breaks caused by the CRISPR-Cas system, ultimately leading to bacterial death or the permanent loss of drug-resistance genes [160]. This process not only eliminates bacterial drug resistance, but also preserves the survival of non-resistant strains, thereby achieving the precise clearance of drug-resistant bacteria. Therefore, Kiga et al. developed CRISPR-Cas13a-based antimicrobials for sequence-specific bacterial killing [161]. The system utilizes guide RNAs (gRNAs) to direct Cas13a to recognize the species/strain-specific RNA of the target bacteria; upon binding, Cas13a’s activated ribonuclease activity cleaves both the target and collateral RNAs, disrupting essential cellular processes and inducing bacterial death. Experimental validation confirmed its efficacy in killing target bacteria in vitro and high specificity with minimal impact on non-target strains, offering a novel “precision antibacterial” strategy to address antibiotic resistance, distinct from broad-spectrum antibiotics. In practical applications, the delivery of the CRISPR-Cas system mainly relies on three types of vectors: plasmid vectors, phage vectors, and nanoparticle vectors [162]. By designing sgRNA that matches the conserved sequences of drug-resistance genes and combining it with efficient vector delivery, the CRISPR-Cas system can achieve precise strikes against drug-resistant bacteria, opening up new avenues for solving the global problem of AMR.

### 3.5. Other Antimicrobial Therapies

Phage therapy, probiotic therapy, antimicrobial peptides (AMPs), and antimicrobial nanoparticle technology represent innovative strategies leveraging biological or nanoscale mechanisms to combat MDR bacteria. Phage therapy utilizes lytic phages, whose endolysins degrade bacterial peptidoglycan, inducing osmotic lysis. Their host specificity and exopolysaccharide depolymerases (encoded in tail fibers) enable biofilm penetration, synergizing with antibiotics to eliminate embedded pathogens like *A. baumannii* [163,164,165,166,167]. While advantageous for rapid development and low toxicity, narrow spectra and immunogenicity require optimization, with bacterial CRISPR-Cas and phage anti-CRISPR proteins driving evolutionary adaptations [168].

Probiotic therapy relies on bacteriocins—short peptides from beneficial microbes—that disrupt pathogenic cell walls (Gram-positive) or interfere with gene expression (Gram-negative) [169]. Lanthipeptides like nisin inhibit MRSA and vancomycin-resistant *enterococci*, while *Lactobacillus gasseri* OLL2716 targets drug-resistant *Helicobacter pylori* [170,171]. Genetically modified probiotics, such as engineered *E. coli* Nissle 1917, enhance efficacy against *P. aeruginosa* infections [172].

Therapeutics based on AMPs exert broad-spectrum activity via cell wall/membrane disruption (e.g., teixobactin binding Lipid II), macromolecule synthesis inhibition, and immune modulation [173,174,175,176,177,178]. Synthetic AMPs (e.g., SAAP-148) address natural AMP limitations (toxicity, protease degradation) and show promise against MDR strains [179,180,181,182]. Antimicrobial nanoparticles (e.g., AgNPs) act through membrane disruption, ion release, reactive oxygen species (ROS) production, and DNA interference, with biocompatible modifications (chitosan, titanium dioxide) reducing cytotoxicity [183,184,185,186].

Antisense therapy, EPIs, and metabolic promoters target bacterial resistance mechanisms at the molecular level. Antisense oligonucleotides (PNAs, PMOs) silence resistance genes via RNA silencing or ribonuclease H1-mediated mRNA degradation, restoring antibiotic sensitivity. Cell-penetrating peptides facilitate delivery, as demonstrated by targeting *mecA* in MRSA to revive β-lactam susceptibility [187,188,189,190,191].

Therapeutics based on EPIs block bacterial efflux pumps, increasing intracellular antibiotic concentrations. Compounds like MC-207,110 (PAβN) enhance levofloxacin activity against *P. aeruginosa* overexpressing Mex pumps [192,193], while N-methylpyrrolidine boosts ofloxacin efficacy in *E. coli* with AcrAB/AcrEF pumps [194,195]. These inhibitors act via competitive binding, with sources spanning natural products and synthetics.

Metabolic promoters awaken dormant persister cells in biofilms, enhancing antibiotic susceptibility. Intermediate metabolites (glucose, pyruvate) induce proton motive force, promoting aminoglycoside uptake [196,197], while cis-2-decenoic acid reactivates *P. aeruginosa* and *E. coli* persisters, synergizing with tobramycin [198]. These strategies address biofilm tolerance, a key driver of chronic infections.

Immunotherapy, anti-virulence therapy, and antimicrobial photodynamic therapy (aPDT) focus on host defense modulation or bacterial pathogenicity rather than direct killing. Immunotherapy uses vaccines and antibodies to curb MDR infections: recombinant vaccines target resistant *S. aureus*, while IgY and monoclonal antibodies (e.g., Raxibacumab) neutralize virulence factors [199,200]. Though no *P. aeruginosa* vaccine is approved, passive immunization reduces bacteremia risk in burn patients.

Anti-virulence therapy weakens pathogenicity by inhibiting virulence factors. Baicalin blocks *S. aureus* α-toxin-mediated cell lysis [201], and LC10 (α-toxin antibody) improves murine survival when combined with antibiotics [202]. Inhibitors of virulence regulators (e.g., glycyrrhetinic acid targeting *S. aureus* SaeR) reduce pathogenicity [203,204].

aPDT generates ROS via photosensitizers and light, damaging bacterial macromolecules. It avoids resistance, with MDR *E. coli* showing increased antibiotic sensitivity post-treatment [205,206,207,208,209]. While effective for superficial infections, enhancing the depth penetration remains a challenge. Together, these strategies offer alternatives to traditional antibiotics, addressing the global resistance crisis through diverse, targeted mechanisms.

Other antimicrobial strategies include drug repurposing, combined therapy, development of known antimicrobial derivatives, and antibiotics targeting bacterial proteins. Drug repurposing, using existing drugs for new antimicrobial uses (e.g., ciclopirox, pentamidine), accelerates discovery [210,211]. Combined therapy, via multi-target mechanisms (e.g., enhancing penetration, inhibiting pathways), boosts efficacy and delays resistance [212,213,214,215,216]. Derivatives of known agents (e.g., tigecycline, cefidericol) and resistance inhibitors (e.g., β-lactamase inhibitors) bypass resistance [217,218]. Antibiotics targeting bacterial proteins, aided by genomics, face challenges like membrane penetration, with no clinical success yet [219,220]. These strategies address multidrug resistance, bridging clinical needs and innovation.

## 4. Closing Remarks

The discovery of antibiotics marked a turning point in modern medicine and has had a profound impact on human health. However, their extensive use in both the medical and livestock industries has given rise to the increasingly severe problem of antibiotic resistance, particularly the emergence of superbugs, which has become a major global public health challenge. The mechanisms of bacterial resistance are complex and mainly involve three types: intrinsic resistance, acquired resistance, and adaptive resistance. Specific manifestations include, but are not limited to, target mutation or modification rendering antibiotics ineffective; changes in cell membrane permeability or activation of efflux pumps reducing drug concentration; synthesis of enzymes such as hydrolases or modifying enzymes that inactivate drugs; the production of protective proteins that hinder drug action; acquisition of resistance genes through HGT; reduction in outer membrane porins or alteration of pore size to decrease drug uptake; and the formation of biofilms that act as physical barriers.

In the strategies to combat bacterial resistance, TCM antibacterial enhancers demonstrate advantages in natural origin, multi-target mechanisms, and low resistance induction. When combined with existing antibiotics, they can rapidly reverse resistant phenotypes. However, their clinical application is limited by challenges such as complex composition, standardization difficulties, and unclear mechanisms [136]. QSIs reduce bacterial pathogenicity by interfering with cell–cell communication, making them suitable for adjunctive therapy in chronic biofilm infections. Nevertheless, they cannot completely eradicate bacteria and carry risks of bacterial escape [221]. The MSW-based strategy leverages pharmacokinetic/pharmacodynamic optimization for clinical implementation without requiring new drug development, serving as a “core lever” in antimicrobial stewardship. Only a few antibiotics have MPC doses exceeding safe limits, restricting their use [222]. Meanwhile, CRISPR-Cas systems offer precise elimination of resistant bacteria with novel mechanisms, but face low delivery efficiency, significant ethical/regulatory barriers, and limited translational value, keeping them confined to basic research stages [189]. Among the other antimicrobial approaches, phage therapy is effective against persistent MDR infections but suffers from narrow spectra and poor immune neutrality, limiting broad applicability [223]. AMPs and nanoparticles exhibit low resistance potential but encounter stability issues, high costs, and pending safety evaluations. Anti-virulence therapy exerts minimal selection pressure but fails to eradicate pathogens. EPIs can restore antibiotic sensitivity but must overcome toxicity and spectrum challenges [192]. Immunotherapy, as a preventive strategy, holds high translational value but is hampered by difficulties in vaccine development against highly variable pathogens.

In summary, the current strategies to combat bacterial resistance can be broadly categorized into two types: one that focus on enhancement and management approaches with high translational potential and closer clinical applicability (such as TCM synergists, MSW medication optimization, and QSIs), which offer the advantage of rapid integration with existing antibiotics to enhance efficacy and delay resistance development; the other comprises cutting-edge technologies with novel mechanisms but significant technical challenges (e.g., CRISPR-Cas, phage therapy, and AMPs), requiring breakthroughs in delivery efficiency, safety, and standardization. Therefore, it is likely that no single strategy can address all challenges at present. In the future, a precise combination and co-application of these strategies could be tailored to different infection types and clinical demands, ultimately providing a broader blueprint for human and animal health.

## Data Availability

No new data were created or analyzed in this study.
